# Quercetin Represses Apolipoprotein B Expression by Inhibiting the Transcriptional Activity of C/EBPβ

**DOI:** 10.1371/journal.pone.0121784

**Published:** 2015-04-15

**Authors:** Makoto Shimizu, Juan Li, Jun Inoue, Ryuichiro Sato

**Affiliations:** Department of Applied Biological Chemistry, The University of Tokyo 1-1-1 Yayoi, Bunkyo, Tokyo, Japan; University of Massachusetts Medical, UNITED STATES

## Abstract

Quercetin is one of the most abundant polyphenolic flavonoids found in fruits and vegetables and has anti-oxidative and anti-obesity effects. Because the small intestine is a major absorptive organ of dietary nutrients, it is likely that highly concentrated food constituents, including polyphenols, are present in the small intestinal epithelial cells, suggesting that food factors may have a profound effect in this tissue. To identify novel targets of quercetin in the intestinal enterocytes, mRNA profiling using human intestinal epithelial Caco-2 cells was performed. We found that mRNA levels of some apolipoproteins, particularly apolipoprotein B (apoB), are downregulated in the presence of quercetin. On the exposure of Caco-2 cells to quercetin, both mRNA and protein levels of apoB were decreased. Promoter analysis of the human *apoB* revealed that quercetin response element is localized at the 5′-proximal promoter region, which contains a conserved CCAAT enhancer-binding protein (C/EBP)-response element. We found that quercetin reduces the promoter activity of *apoB*, driven by the enforced expression of C/EBPβ. Quercetin had no effect on either mRNA or protein levels of C/EBPβ. In contrast, we found that quercetin inhibits the transcriptional activity of C/EBPβ but not its recruitment to the apoB promoter. On the exposure of Caco-2 cells to quercetin 3-*O*-glucuronide, which is in a cell-impermeable form, no notable change in apoB mRNA was observed, suggesting an intracellular action of quercetin. *In vitro* interaction experiments using quercetin-conjugated beads revealed that quercetin binds to C/EBPβ. Our results describe a novel regulatory mechanism of transcription of apolipoprotein genes by quercetin in the intestinal enterocytes.

## Introduction

Quercetin is one of the most abundant flavonoids present in a wide variety of fruits and vegetables, including onions, and has several beneficial properties against metabolic diseases through anti-oxidative and anti-inflammatory effects [1]. Several studies demonstrated that oxidative stress and other reactive oxygen species play important roles in the development of cancer, neurodegenerative disease, and diabetes. Among polyphenolic flavonoids, quercetin and luteolin show the strongest anti-oxidative activity. Moreover, the anti-diabetic effects of quercetin against type 1 [2] and type 2 [3] diabetes have been reported. Although quercetin exerts beneficial effects on metabolic diseases, few molecular targets of quercetin have been identified. The small intestine is a major absorptive tissue of dietary nutrients. After their intestinal absorption, the flavonoids among them are likely to be conjugated and excreted efficiently in the urine, resulting in a lower accumulation of quercetin in the body [4–6]. Because concentrated food constituents may be present in the small intestinal epithelial cells, food factors may exert a more profound effect in this tissue compared with other organs.

In this study, we performed mRNA profiling to identify novel targets of quercetin in the intestinal enterocytes. We found that quercetin reduces the gene expression of apolipoproteins, including apolipoprotein B (apoB), in the human intestinal epithelial Caco-2 cells. Both mRNA and proteins of apoB were decreased by the quercetin treatment. Promoter analysis of the human *apoB* shows that a CCAAT enhancer-binding protein(C/EBP)-response element is important for the quercetin action. Futhermore, we found that quercetin binds C/EBPβ protein, thereby inhibiting its transcriptional activity. These results demonstrate a novel mechanism underlying the transcriptional regulation of apolipoprotein genes by quercetin in the small intestine.

## Materials and Methods

### Materials

Quercetin was obtained from Jena Bioscience. Quercetin 3-*O*-glucuronide and the protease inhibitor cocktail were obtained from Sigma.

### Antibodies

Anti-apoB antibody (ab7616) was obtained from Abcam, anti-β-actin antibody (A5441) was obtained from Sigma, anti-C/EBPβ antibody (sc-150) was obtained from Santa Cruz Biotechnology, and anti-lamin antibody (2032) was obtained from cell signaling technology.

### Cell culture

Caco-2 cells were maintained in medium A (Dulbecco’s modified Eagle’s medium supplemented with 10% fetal bovine serum, non-essential amino acids, 100 units/mL penicillin, and 100 μg/mL streptomycin) at 37°C in an atmosphere containing 5% CO_2_. Except for transfection experiments and chromatin immunoprecipitation (ChIP) assays, cells that had been cultured for 14 days after confluency were considered differentiated. HepG2 cells were maintained in medium B (Dulbecco’s modified Eagle’s medium supplemented with 10% fetal bovine serum, 100 units/mL penicillin, and 100 μg/mL streptomycin) at 37°C in an atmosphere containing 5% CO_2_. Caco-2 cells and HepG2 cells were purchased from ATCC.

### Plasmids

Luciferase reporter plasmids for human apoB promoters were constructed using KpnI/BglII PCR fragments coding for the indicated 5′-untranslated regions of human *apoB* into a pGL3-Basic vector (Promega). A luciferase reporter plasmid for C/EBP-response element (C/EBP-RE) was constructed using a double-stranded DNA coding four copies of C/EBP-RE of mouse C/EBPα promoter into a pGL3 promoter vector containing the promoter for simian virus 40 (SV40) (Promega). An expression plasmid for GAL4-hC/EBPβ (amino acid residues 1–345) was constructed by inserting a PCR fragment, which encodes human C/EBPβ into an expression plasmid for the DNA-binding domain of the yeast transcription factor GAL4 (pCMX-GAL4).

An expression plasmid for pcDNA-hC/EBPβ was provided by Dr. Hidetoshi Hayashi. A reporter plasmid for yeast GAL4 (TK-MH100x4-Luc) and an expression plasmid for pCMX-GAL4 were provided by Drs. David J. Mangelsdorf and Steven A. Kliewer.

### Co-transfection and luciferase assay

Caco-2 cells were placed in a 12-well plate at a density of 1.5 × 10^5^ cells/well, cultured with medium A for 24 h, and then transfected with 500 ng of one of the reporter plasmids and 500 ng of pCMV-β-Gal, an expression plasmid for β-galactosidase, using the calcium phosphate method. For co-transfection experiments, 250 ng of one of the reporter plasmids, 250 ng of pCMV-β-Gal, and 500 ng of each of the expression plasmids were added. Twenty four hours after transfection, medium was replaced with medium A and vehicle or the indicated compounds were added. After incubation for an additional 24 h, the luciferase and β-galactosidase activities were determined, as described previously [7]. Normalized luciferase values were determined by dividing the luciferase activity by the β-galactosidase activity.

### Western blot analysis

Caco-2 cells were differentiated by continuous culture for 14 days after they reached confluency. After treatment, cells were harvested, and western blot analysis was performed, as described previously [8].

### Real time PCR

Total cell RNA was extracted using ISOGEN (NIPPON GENE) according to the manufacturer’s instructions. The high capacity cDNA reverse transcription kit (Applied Biosystems) was used to synthesize and amplify cDNA from total RNA. Quantitative real-time PCR (SYBR green and TaqMan) was performed using an Applied Biosystems 7000 sequence detection system. Relative mRNA levels were determined by normalizing to the 36B4 transcript. The sequences of the primer sets used in this study were as follows: 36B4 [9], 5′-TGCATCAGTACCCCATTCTATCA-3′ and 5′-AAGGTGTAATCCGTCTCCACAGA-3′; apoB [10], 5′-GCCATTGCGACGAAGAAAATA-3′ and 5′-TGACTGTGGTTGATTGCAGCTT-3′; apoCII, 5′-TGGGAGTCAGCAAAGACA-3′ and 5′-GCCTGTGTAAGTGCTCATG-3′; apoCIII, 5′-GCTCAGTTCATCCCTAGA-3′ and 5′-CGGCCTCTGAAGCTCG-3′; apoE, 5′-TGCGTTGCTGGTCACATTC-3′ and 5′-TCTGTCTCCACCGCTTGCT-3′; and MTP, 5′-TGTGCTTTTTCTCTGCTTCATTTC-3′ and 5′-GCTTGTACAGCCGGTCATTATTT-3′. The TaqMan ID number for apoA-I is Hs00163641_m1.

### ChIP assay

Caco-2 cells were grown in 10-cm dishes with medium A to confluency, followed by treatment with vehicle or 100 μM quercetin for 10 h. The cells were then processed for ChIP assay using a reagent kit (Millipore), as recommended by the manufacturer. Immunoprecipitation was performed with normal rabbit IgG (Santa Cruz Technology) or an anti-C/EBPβ antibody (Santa Cruz Technology). Real time PCR used the following primers: human apoB C/EBP-RE, 5′-CTTCAAGGCTCAAAGAGAAGCC-3′ and 5′-AGGTCCCGGTGGGAATG-3′ and human apoB distal region, 5′-GGGCACAGTTCCATCTACAA-3′ and 5′-CCTATCTCGTTTCTGCCTATGAC-3′.

### Binding assay using quercetin-conjugated beads

Quercetin-conjugated agarose beads and their control beads were provided from RIKEN NPDepo [11]. After Caco-2 cells were treated with vehicle or 100 μM quercetin for 12 h, cells were collected with the binding buffer [10 mM Tris-HCl (pH 7.6), 50 mM KCl, 5 mM MgCl_2_, 1 mM EDTA, and a protease inhibitor cocktail]. Cell lysates (1 mg of protein) were precleared by control beads (10 μl) and then incubated with quercetin beads or control beads (15 μl) for 12 h at 4°C. The reacted beads were washed with the binding buffer, and co-precipitated proteins were detected by western blot analysis.

### Statistical analysis

All results are presented as mean ± standard deviation (±SD) and evaluated using the Student’s *t*-test. Significance was assumed at p < 0.05 and p < 0.01.

## Results

### Expression of apolipoproteins is decreased by quercetin

**Fig 1:**
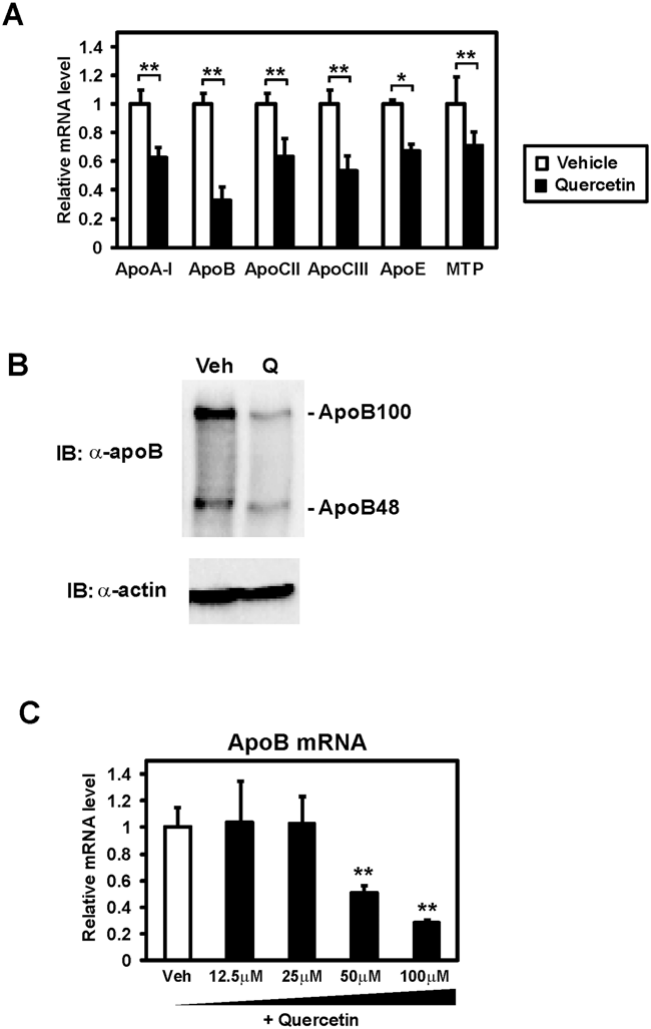
Quercetin repressed the expression of genes related to lipoprotein metabolism in Caco-2 cells. (**A**) Differentiated Caco-2 cells were treated with vehicle or 100 μM quercetin for 24 h, after which total RNA was isolated. mRNA levels, which were determined by quantitative real-time PCR, are presented as relative expression after normalization to 36B4 mRNA. Relative mRNA levels in vehicle-treated cells were set to 1. Data are presented as mean ± SD (n = 3). *p < 0.05, **p < 0.01. (**B**) Differentiated Caco-2 cells were treated with vehicle (Veh) or 100 μM quercetin (Q) for 12 h. Proteins were extracted and immunoblots (IB) were performed. (**C**) Differentiated Caco-2 cells were exposed to vehicle or the indicated doses of quercetin for 24 h, after which total RNA was isolated. Data are presented as mean ± SD (n = 3). **p < 0.01.

Human intestinal epithelial Caco-2 cells were used to identify novel targets of quercetin in the intestinal enterocytes. Total RNA was prepared from differentiated Caco-2 cells cultured with 100 μM quercetin for 24 h. Then mRNA levels of several genes related to lipid metabolism were analyzed by quantitative real time PCR. This mRNA profiling revealed that the gene expression of some apolipoproteins, including apolipoprotein A-I (apoA-I) and apoB, was significantly decreased by quercetin (Fig 1A). In addition, we observed downregulation of gene expression of microsomal triglyceride transfer protein (MTP), which has a crucial role in chylomicron and very low density lipoprotein (VLDL) assembly. Because apoB expression was markedly suppressed by quercetin, we decided to focus on further analyzing the regulation of apoB gene expression. Exposure of quercetin for 12 h was enough to suppress apoB expression (S1 Fig). Caco-2 cells synthesize both apoB48 and apoB100, although they are produced *in vivo* in the intestine and liver, respectively [12]. Western blot analyses revealed that both intracellular apoB48 and apoB100 proteins were decreased by the quercetin treatment for 12 h (Fig 1B). ApoB expression was decreased at 50 and 100 μM quercetin (Fig 1C). These results indicate that quercetin reduces apoB expression both in mRNA and protein levels.

### A C/EBP-response element in the ApoB promoter mediates quercetin action

**Fig 2:**
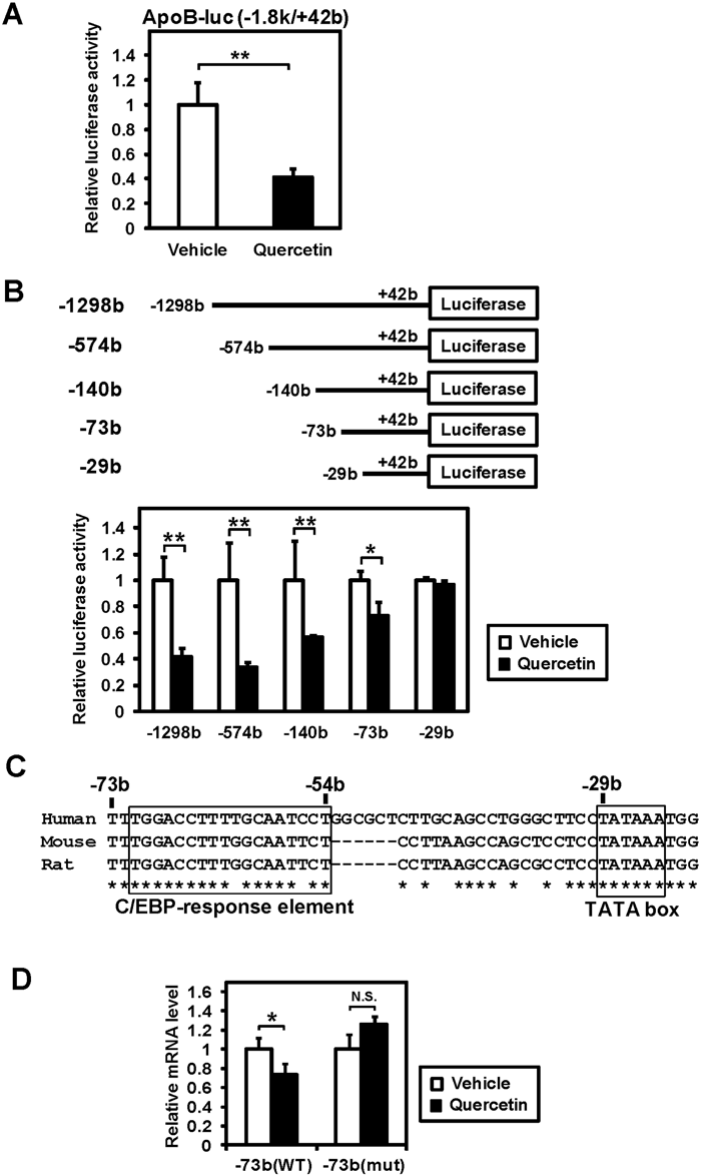
Identification of the quercetin-response element of the human apoB promoter. (**A, B, D**) Caco-2 cells were transfected with the reporter gene, which contains the indicated upstream region of human *apoB* and an expression plasmid for β-galactosidase. After transfection, cells were exposed to vehicle or 100 μM quercetin for 24 h. Luciferase activities were normalized to β-galactosidase activities; a value of 1 was set in the absence of quercetin. Data are presented as mean ± SD (n = 3). *p < 0.05, **p < 0.01, N.S., not significant. (**C**) DNA sequences of the human apoB (NT_022184), mouse apoB (NC_000078), and rat apoB (NW_047758) promoters around the quercetin-response element. Conserved DNA sequences are marked by asterisks.

To investigate the mechanism underlying the quercetin-mediated suppression of apoB expression, reporter gene assays were performed. We generated a reporter gene construct located in the 1.8k upstream region of the human *apoB*. When Caco-2 cells were treated with quercetin, the promoter activity of the 1.8k upstream region was significantly decreased (Fig 2A). To determine the precise region of the quercetin-response element, we next generated a series of deletion constructs of the upstream region of *apoB*. Reporter gene assays showed that the promoter activity suppressed by quercetin was not affected by deleting the region between −1298 bp and −73 bp, even though the repression of the promoter activity by quercetin slightly diminished when the region between −574 bp and −73 bp was deleted (Fig 2B). In contrast, the inhibitory effect of quercetin on the promoter activity was completely abolished when the region between −73 bp and −29 bp was deleted, indicating that this region includes the quercetin-response element (Fig 2B). The DNA sequence analysis using CLUSTALW alignment software revealed that the region between −71 bp and −54 bp was highly conserved among species. This DNA sequence was previously reported as the CCAAT enhancer-binding protein (C/EBP)-response element [13–15] (Fig 2C). The suppression of the promoter activity caused by quercetin was abolished when the C/EBP-response element sequence was mutated (Fig 2D). These results suggest that quercetin repressed transcription of *apoB* through C/EBP.

### Quercetin inhibits the C/EBPβ-mediated activation of the apoB promoter

**Fig 3:**
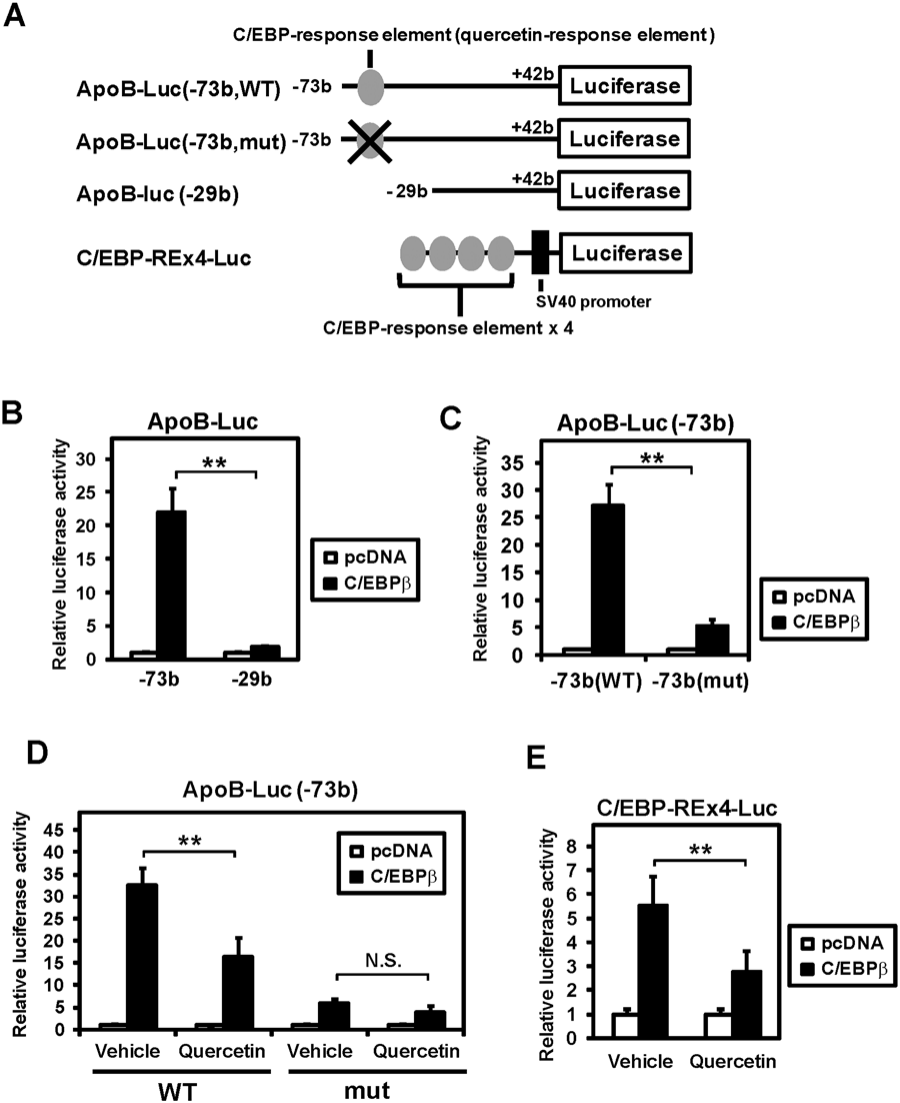
Quercetin inhibits the C/EBPβ-mediated activation of the human apoB promoter. (**A**) A map of the reporter plasmids was used. Gray circles, C/EBP-response element; closed box, SV40 promoter. (**B,C**) Caco-2 cells were transfected with the indicated reporters and expression plasmids for β-galactosidase and C/EBPβ. Luciferase activities were normalized to β-galactosidase activities; a value of 1 was set in the absence of C/EBPβ. Data are presented as mean ± SD (n = 3). **p < 0.01. (**D,E**) Caco-2 cells were transfected with the indicated reporters and expression plasmids for β-galactosidase and C/EBPβ. After transfection, cells were treated with vehicle or 100 μM quercetin for 24 h. Luciferase activities were normalized to β-galactosidase activities; a value of 1 was set in the absence of C/EBPβ. Data are presented as mean ± SD (n = 3). **p < 0.01, N.S., not significant.

**Fig 4:**
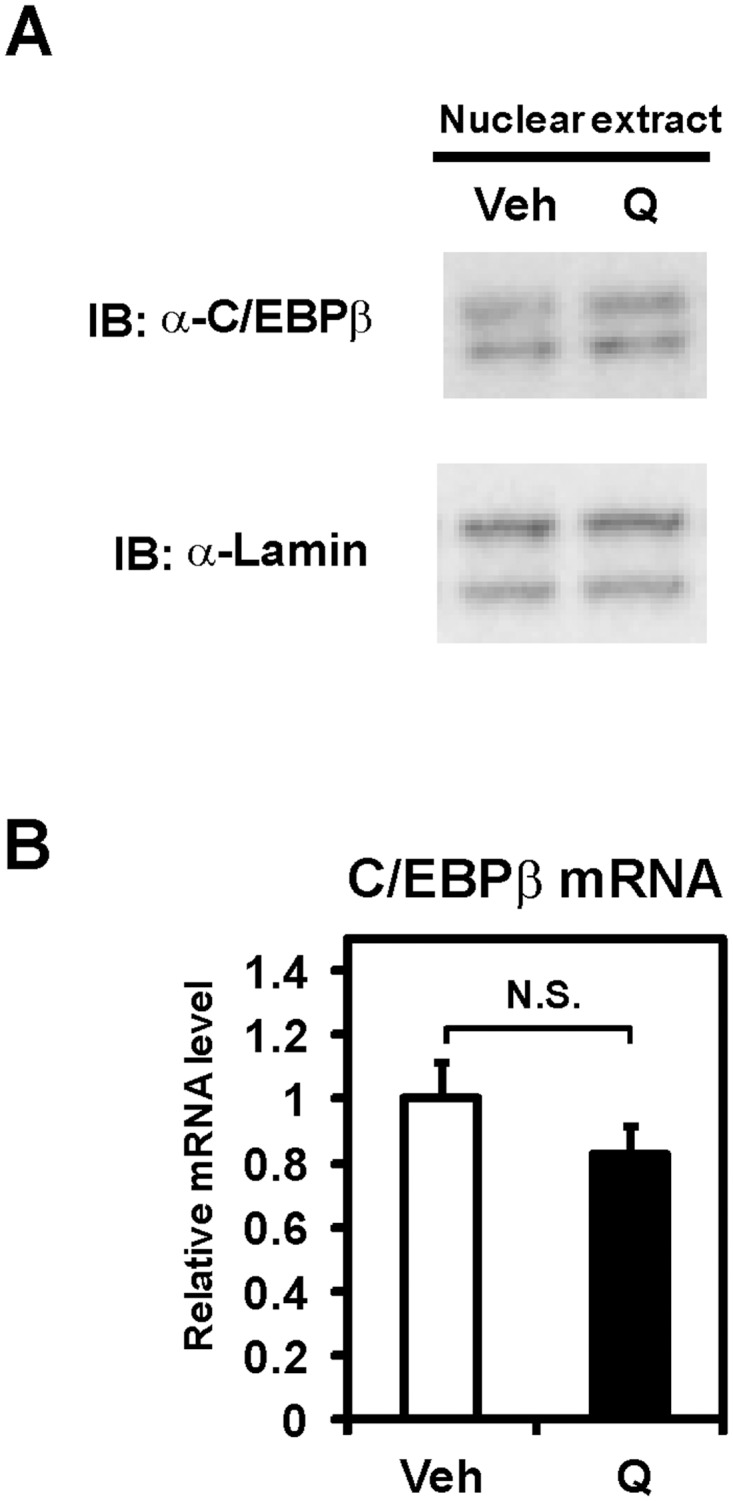
C/EBPβ expression was not altered by quercetin. (**A**) Immunoblot (IB) analysis used nuclear extracts from differentiated Caco-2 cells treated with vehicle (Veh) or 100 μM quercetin (Q) for 12 h. (**B**) Differentiated Caco-2 cells were exposed to vehicle (Veh) or 100 μM quercetin (Q) for 12 h, after which total RNA was isolated. mRNA levels, which were determined by quantitative real-time PCR, are presented as relative expression after normalization to 36B4 mRNA. Relative mRNA levels in vehicle-treated cells were set to 1. Data are presented as mean ± SD (n = 3). N.S., Not significant.

Because C/EBPβ regulates the intestinal expression of *apoB* among C/EBP family proteins [16], we investigated the effect of C/EBPβ on the quercetin-response element. Reporter gene assays using C/EBPβ expression plasmid were performed. When C/EBPβ was expressed in Caco-2 cells, the promoter region between −73 bp and +42 bp was strongly activated (−73 bp, Fig 3B). In contrast, deletion of the quercetin-response element (−29 bp, Fig 3B) or mutating the C/EBP-response element (Fig 3C) strongly reduced the promoter activation by C/EBPβ. Furthermore, we examined whether quercetin represses the C/EBPβ-mediated activation of the apoB promoter. Reporter gene assay showed that the stimulation of the apoB promoter by C/EBPβ was significantly reduced in the presence of quercetin (WT, Fig 3D). However, a mutation in the C/EBP-response element abolished the suppression of C/EBPβ-driven promoter activation by quercetin (mut, Fig 3D). To confirm a common inhibitory role of quercetin on C/EBPβ target genes, reporter gene assay using the artificial promoter containing four copies of C/EBP-response elements derived from the mouse C/EBPα promoter (C/EBP-REx4-Luc, Fig 3A) was performed. Similar to the apoB promoter, quercetin repressed the C/EBPβ-mediated activation of C/EBP-REx4-Luc (Fig 4D). These results indicate that quercetin inhibits the promoter activation mediated by C/EBPβ.

### C/EBPβ expression is not affected by quercetin

Because quercetin inhibits the induction of C/EBPβ protein in response to the proteasome inhibitors [17], we hypothesized that quercetin regulates apoB transcription through decreased C/EBPβ proteins. To investigate the protein levels of C/EBPβ, nuclear extracts were prepared from Caco-2 cells treated with quercetin for 12 h. Western blot analyses showed no detectable changes of nuclear C/EBPβ proteins by quercetin (Fig 4A). Quantitative real time PCR analyses similarly revealed that quercetin has no effect on the mRNA level of C/EBPβ (Fig 4B). These results indicate that quercetin does not affect C/EBPβ expression in Caco-2 cells.

### Transcriptional activity, but not DNA-binding activity, of C/EBPβ is inhibited by quercetin

**Fig 5:**
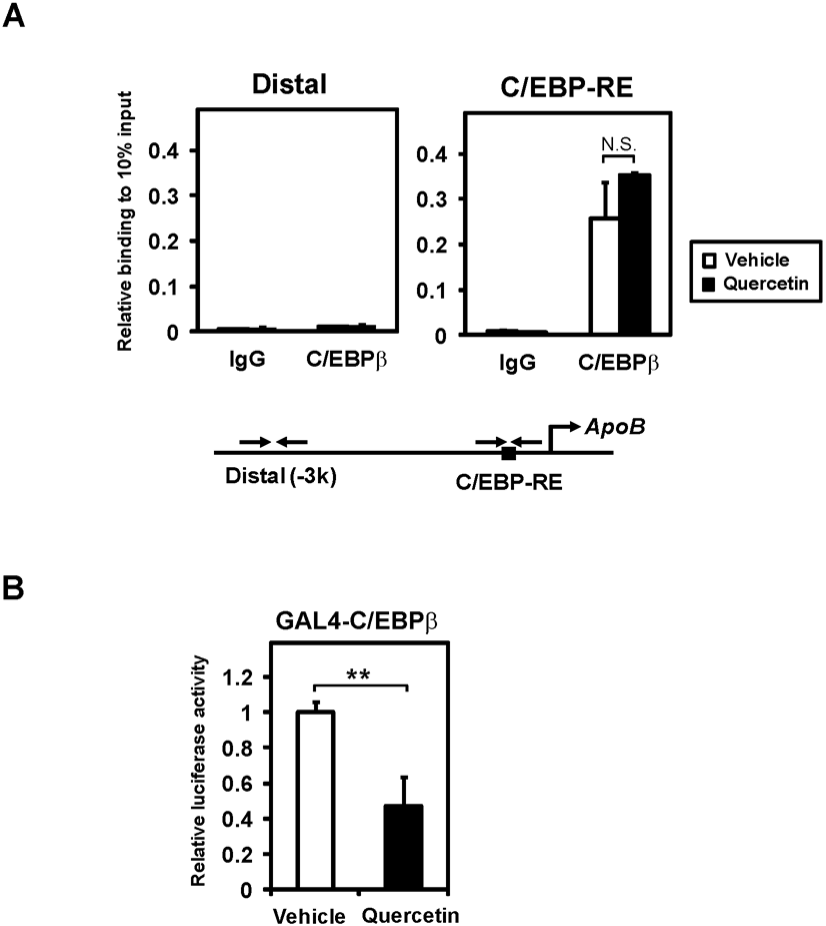
Quercetin represses the transcriptional activity, but not DNA-binding activity, of C/EBPβ. (**A**) Caco-2 cells treated with vehicle or 100 μM quercetin for 10 h were analyzed by ChIP assays using an antibody against C/EBPβ or control IgG. Bound DNA was quantified by real-time PCR using specific primer sets for the C/EBP-RE region or distal control region. Data are presented as mean ± SD (n = 3). N.S., Not significant. (**B**) HEK293 cells were transfected with the reporter gene containing four copies of a yeast GAL4 upstream activation sequence (TK-MH100x4-luc) and expression plasmids for β-galactosidase and C/EBPβ fused with the yeast GAL4 DNA-binding domain (GAL4-C/EBPβ). After transfection, cells were exposed to vehicle or 100 μM quercetin for 24 h. Luciferase activities were normalized to β-galactosidase activities; a value of 1 was set in the absence of quercetin. Data are presented as mean ± SD (n = 3). **p < 0.01.

We next performed ChIP assays to examine the DNA-binding activity of C/EBPβ. Caco-2 cells were exposed to 100 μM quercetin for 10 h, and then chromatin was prepared. To isolate the C/EBPβ-bound genomic DNA, a chromatin immunoprecipitation by C/EBPβ antibody was performed. Quantitative real time PCR was used to detect the recruitment of C/EBPβ to the promoter using primer sets covering the region containing the C/EBP-response element (C/EBP-RE) or the upstream control region, which lacked C/EBP-RE (−3 kb, distal) of *apoB*. We observed that C/EBPβ was selectively recruited to C/EBP-RE but not the distal region (Fig 5A). This DNA-binding of C/EBPβ was not decreased in the presence of quercetin, suggesting that quercetin reduces apoB expression, independent of the DNA-binding activity of C/EBPβ.

We next examined the transcriptional activity of C/EBPβ using a fusion construct containing human C/EBPβ and the DNA-binding domain of yeast transcription factor GAL4 (GAL4-hC/EBPβ). The transcriptional activity of this fusion protein was analyzed by the reporter, which contains a GAL4 DNA-binding target sequence (TK-MH100x4-Luc). As shown in Fig 5B, quercetin significantly reduced the activity of GAL4-C/EBPβ in HEK293 cells. These results indicated that quercetin directly inhibits the transcriptional activity but not the DNA-binding activity of C/EBPβ.

### Quercetin binds to C/EBPβ

**Fig 6:**
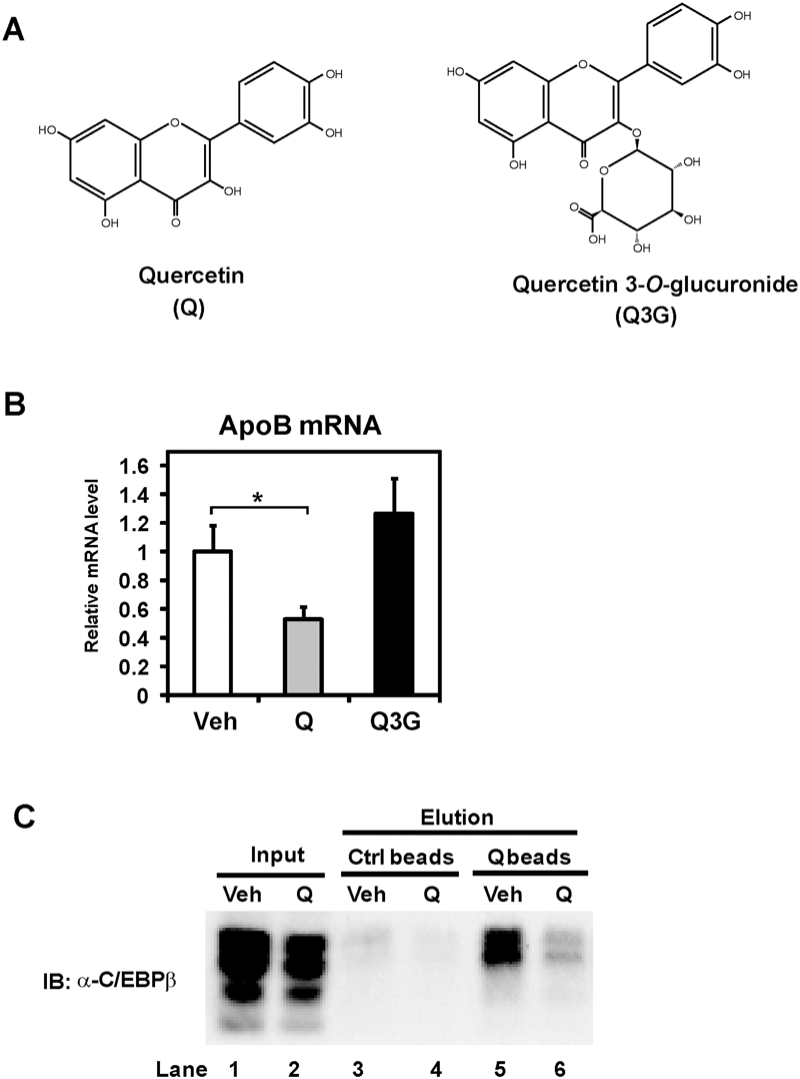
Intracellular quercetin binds to C/EBPβ. (**A**) Chemical structures of quercetin and quercetin 3-*O*-glucuronide. (**B**) Differentiated Caco-2 cells were treated with vehicle (Veh) or 100 μM quercetin (Q) or 100 μM quercetin 3-*O*-glucuronide (Q3G) for 24 h, after which total RNA was isolated. mRNA levels, which were determined by quantitative real-time PCR, are presented as relative expression after normalization to 36B4 mRNA. Relative mRNA levels in vehicle-treated cells were set to 1. Data are presented as mean ± SD (n = 3). *p < 0.05. (**C**) Caco-2 cells were exposed to vehicle (Veh) or 100 μM quercetin (Q) for 12 h, and cell lysates were prepared. Pre-cleared lysates with control beads were incubated with control (Ctrl) beads or quercetin (Q)-conjugated beads. The reacted beads were washed, and the coprecipitated proteins were detected by immunoblot (IB) analysis.

We next examined whether quercetin affected the C/EBPβ transcriptional activity intracellularly. Quercetin 3-*O*-glucuronide (Fig 6A) is not absorbed efficiently compared with quercetin aglycone [18]. Caco-2 cells were treated with these flavonoids for 24 h, and then mRNA was prepared for mRNA analysis. We observed that quercetin aglycone, but not quercetin 3-*O*-glucuronide, reduced apoB mRNA (Fig 6B), indicating the intracellular action of quercetin. Because significant amount of quercetin could be found in the nucleus [19], we hypothesized that quercetin directly works on nuclear C/EBPβ. To confirm the binding of quercetin to C/EBPβ, experiments using quercetin-immobilized agarose beads (quercetin beads) [11] were performed. Caco-2 cells were cultured in the presence of 100 μM quercetin for 12 h. Lysates prepared from Caco-2 cells were incubated with quercetin beads or equivalent control beads, and precipitated proteins with beads were detected by C/EBPβ antibody. Western blot analysis revealed that endogenous C/EBPβ proteins were coprecipitated with quercetin beads, whereas no associations with control beads were detected (Fig 6C, Lane 3 and 5). Moreover, the quercetin treatment reduced the C/EBPβ binding to quercetin beads, suggesting that C/EBPβ prepared from the cells incubated with quercetin was no longer able to associate with quercetin beads (Fig 6C, Lane 5 and 6). These results showed that quercetin binds to C/EBPβ after uptake by cells and represses the transcriptional activity of C/EBPβ.

## Discussion

ApoB is a major apolipoprotein synthesized in the liver and small intestine and has an important role in the assembly and secretion of hepatic VLDL and intestinal chylomicrons. Overproduction of VLDL observed in insulin resistant states may lead to hypertriglyceridemia and atherosclerosis. In addition, plasma levels of chylomicrons are elevated in type 2 diabetes [20]. Along with apoB, MTP is also essential for the assembly of chylomicrons. Studies using the intestine specific MTP knockout mice [21] and intestine selective MTP inhibitors [22] demonstrated that a loss of MTP function in the small intestine improves the plasma lipid parameters. These observations suggest that an inhibition of intestinal chylomicron production may contribute to medical therapy for obesity and diabetes. Our present study revealed that quercetin reduces expression of several genes related to lipoprotein metabolism, including apoB and MTP (Fig 1A), indicating an effective inhibition of chyromicron formation by quercetin. Previous studies reported C/EBP-response elements in the promoter region of some apolipoprotein genes [23], suggesting that quercetin regulates the expression of these genes by inhibiting C/EBPβ. In addition, quercetin represses the secretion of apoB in the enterocytes [24] and hepatocytes [25]. Furthermore, anti-obesity and anti-diabetic effects of quercetin have been reported [2,26]. These studies support the physiological relevance of our present results. Future *in vivo* studies may be required to verify the reduction of intestinal apolipoprotein expression and chylomicron production by quercetin.

ApoB expression is regulated by several steps, such as transcription, mRNA editing, and proteosomal degradation. Hepatocyte nuclear factor 3 (HNF3), HNF4, and C/EBP are the key transcriptional regulators of *apoB* [13]. Hepatic C/EBPα binds to the C/EBP response element of the apoB promoter [27], whereas it is unknown which C/EBP subtype binds to this sequence in the small intestine. In addition to the proximal promoter, intestinal apoB expression requires the intestinal enhancer in the upstream region. It has been reported that HNF4, HNF3β, and C/EBPβ bind to this enhancer region [28]. These prompted us to investigate C/EBPβ association to the C/EBP-response element in the proximal apoB promoter. Our present study revealed that endogenous C/EBPβ binds to the promoter region in Caco-2 cells (Fig 5A). However, these results do not rule out the possibility of involvement of other C/EBP family members. Previous studies reported that some C/EBP subtypes, including C/EBPα are also expressed in the small intestine [29]. Moreover, we observed that quercetin represses the C/EBPα-mediated activation of the apoB promoter in Caco-2 cells by the reporter gene assay (S2 Fig). Further studies including the analysis of transcriptional regulation of *apoB* by other C/EBP subtypes are also needed.

Our results clearly show that the C/EBP-response element between −71 bp and −54 bp is crucial for quercetin-mediated repression of the apoB promoter (Figs 2D and 3D). Quercetin potently inhibited the promoter activity when the region between −574 bp and +42 bp was present. However, deleting the region between −574 bp and −73 bp resulted in the loss of inhibition although quercetin significantly, though not strongly, repressed the promoter activity (Fig 2B). In addition, quercetin reduced apoB-luc promoter activity of (−574 bp), even when the C/EBP-response element was mutated (S3 Fig). These results indicate that the unknown transcription factors other than C/EBP, which appear to bind the region between −574 bp and −73 bp, are also involved in quercetin-mediated repression of apoB gene expression. However, when C/EBPβ was exogenously expressed, quercetin-mediated repression of both the longer and shorter promoter activities was completely abolished by C/EBP-RE mutation (−73 bp mut and −574 bp mut in Fig 3D and S4 Fig, respectively). Therefore, it is clear that the C/EBP-response element located between −71 bp and −54 bp is responsible for a part of the quercetin-dependent downregulation of apoB gene expression. We observed a weak activation of the mutated promoters by C/EBPβ (Fig 3C and S4 Fig), which may have been caused by another C/EBP-response element located between −53 bp and −33 bp [13].

In addition to the small intestine, C/EBPβ is expressed in other tissues, including the liver [29]. We observed that quercetin suppressed *apoB* expression in the human hepatoma HepG2 cells (S5 Fig). Moreover, quercetin has been reported to inhibit apoB secretion from HepG2 cells [25]. These observations indicate that quercetin may act on apoB expression in the small intestine and liver.

Quercetin inhibits the induction of C/EBP protein [17] and the DNA-binding activity of C/EBPβ [30]. We did not observe these effects in the present study (Figs 4A and 5A), but an inhibition of the transcriptional activity of C/EBPβ by quercetin was demonstrated. It is possible that some distinct experimental conditions may cause the difference [17,30].

Along with C/EBP, HNF4 is also an important regulator of apolipoprotein expression. AMP-activated protein kinase (AMPK) promotes the degradation of HNF4 protein through phosphorylation [31,32]. HNF4 is highly expressed in the liver and small intestine [33], and quercetin is an activator of AMPK [34,35]. From these observations, we hypothesized that quercetin reduces apoB expression through the degradation of HNF4 protein. However, the reporter gene assay showed that quercetin does not inhibit the HNF4-mediated activation of the apoB promoter (S6 Fig), suggesting a selective action of quercetin on C/EBPβ in the small intestine.

**Fig 7:**
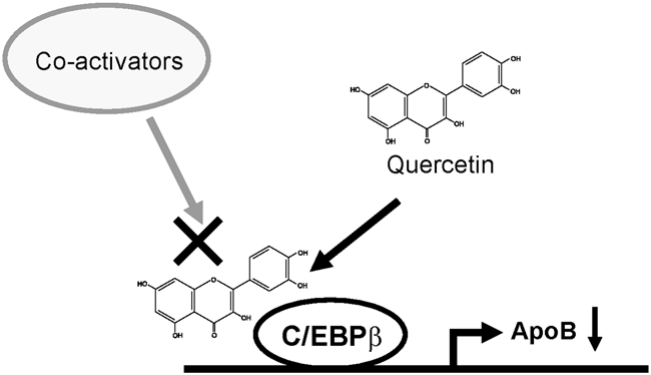
Proposed model for quercetin-mediated repression of apoB expression. Quercetin represses expression of *apoB* through an inhibition of the transcriptional activity of C/EBPβ. Quercetin may interfere in the recruitment of coactivators by binding to C/EBPβ. The present results revealed novel quercetin-mediated regulation of apoB expression.

In conclusion, RNA profiling studies using Caco-2 cells found that quercetin represses the gene expression of apolipoproteins, including apoB. Promoter analysis of the human *apoB* revealed the C/EBP-mediated action of quercetin. Furthermore, studies using GAL4-C/EBPβ and quercetin beads showed that quercetin binds to C/EBPβ and represses its transcriptional activity possibly by inhibiting the recruitment of coactivators (Fig 7). Overall, these results demonstrate a novel function of quercetin as a repressor of the apolipoprotein gene expression through its binding to C/EBPβ protein.

## Supporting Information

S1 Fig Quercetin exposure for 12 h reduces the expression of apoB gene. Differentiated Caco-2 cells were treated with vehicle or 100 μM quercetin for 12 h, after which total RNA was isolated. mRNA levels, which were determined by quantitative real-time PCR, are presented as relative expression after normalization to 36B4 mRNA. Relative mRNA levels in vehicle-treated cells were set to 1. Data are presented as mean ± SD (n = 3). **p < 0.01.
(TIF)

S2 Fig Quercetin inhibits the C/EBPα-mediated activation of the human apoB promoter. Caco-2 cells were transfected with the indicated reporter [apoB-luc (−73b)] and expression plasmids for β-galactosidase and C/EBPα. After transfection, cells were treated with vehicle or 100 μM quercetin for 24 h. Luciferase activities were normalized to β-galactosidase activities; a value of 1 was set in the absence of C/EBPα. Data are presented as mean ± SD (n = 3). *p < 0.05.
(TIF)

S3 Fig Quercetin represses the promoter activity of a mutated reporter containing the −574 bp region of the apoB gene. Caco-2 cells were transfected with the wild-type (WT) or the C/EBP-response element mutated (mut) reporter gene, which contains the indicated upstream region of human *apoB* and a β-galactosidase expression plasmid. After transfection, cells were exposed to vehicle or 100 μM quercetin for 24 h. Luciferase activities were normalized to β-galactosidase activities; a value of 1 was set in the absence of quercetin. Data are presented as mean ± SD (n = 3). *p < 0.05, **p < 0.01.
(TIF)

S4 Fig C/EBP-response element mediates repression of the apoB promoter through C/EBPβ by quercetin. Caco-2 cells were transfected with the indicated reporters and expression plasmids for β-galactosidase and C/EBPβ. After transfection, cells were treated with vehicle or 100 μM quercetin for 24 h. Luciferase activities were normalized to β-galactosidase activities; a value of 1 was set in the absence of C/EBPβ. Data are presented as mean ± SD (n = 3). **p < 0.01, N.S., not significant.
(TIF)

S5 Fig Quercetin reduces apoB expression on hepatocytes. HepG2 cells were treated with vehicle or 100 μM quercetin for 24 h, and the total RNA was isolated. mRNA levels were determined by quantitative real-time PCR, and are presented as relative expression after normalization to 36B4 mRNA. Relative mRNA levels in vehicle-treated cells were set to 1. Data are presented as mean ± SD (n = 3). **p < 0.01.
(TIF)

S6 Fig Quercetin does not inhibit the HNF4α-mediated activation of the human apoB promoter. HEK293 cells were transfected with the indicated reporter [apoB-luc (−73b)] and expression plasmids for β-galactosidase, HNF4α, and C/EBPβ. After transfection, cells were treated with vehicle or 30 μM quercetin for 12 h. Luciferase activities were normalized to β-galactosidase activities; a value of 1 was set in the absence of HNF4α and C/EBPβ. Data are presented as mean ± SD (n = 3). **p < 0.01. N.S., Not significant.
(TIF)
